# Variations in gamma radiation and alpha-emitting radionuclides in correlation with weather and location conditions

**DOI:** 10.1038/s41598-025-10797-2

**Published:** 2025-07-11

**Authors:** D. E. Tchorz-Trzeciakiewicz, J. A. Kamińska

**Affiliations:** 1 Institute of Geological Sciences, University of Wrocław, Pl. M. Borna 9, Wrocław, 50-204 Poland; 2 Department of Applied Mathematics, Wroclaw University of Environmental and Life Sciences, ul. Grunwaldzka 53, Wrocław, 50-357 Poland

**Keywords:** Gamma dose rate, Alpha-emitting radionuclides, Weather conditions, Location conditions, Environmental sciences, Environmental chemistry, Environmental impact, Environmental chemistry, Environmental monitoring

## Abstract

This research examines variations in natural radiation, focusing on terrestrial radiation and radon, in relation to weather and location. Understanding fluctuations in gamma and alpha radiation is crucial for distinguishing meteorologically-induced changes from artificial contamination. Measurements were performed hourly and daily at nine stations over the course of one year. The highest annual gamma doses were recorded in the mountain stations. The lowest gamma radiation levels and alpha-emitting radionuclide concentrations were observed in seaside regions. Precipitation influenced alpha-emitting radionuclide concentrations more than gamma radiation. Hourly variations of gamma radiation showed no consistent pattern. Statistical analysis indicated a positive correlation between gamma radiation and temperature at most locations, except near the sea where sea breezes influenced gamma dose rates. Other meteorological parameters had minimal impact on gamma radiation variations. For alpha-emitting radionuclides, a positive correlation with temperature was found, while atmospheric pressure and wind speed showed negative correlations. Seasonal variations in gamma radiation did not follow a uniform trend across all locations. In the case of seasonal variations in alpha-emitting radionuclides concentrations, the highest average values were observed during autumn and the lowest in spring.

**Supplementary Information:**

The online version contains supplementary material available at 10.1038/s41598-025-10797-2.

## Introduction

Humans are exposed to different kinds of naturally occurring radiation. This includes cosmic radiation, terrestrial radiation and radon, which provide 85.5% of the population’s average dose. The terrestrial component of the natural background is related to the members of the radioactive decay chains of Th-232, U-238 and U-235, along with K-40, which are present in variable quantities in the rocks forming the Earth’s crust. Alpha radiation is considered radiation emitted by radon and radioactive aerosol particles, which constitute decay products of radon and thoron.

Gamma and alpha radiation research is essential for understanding environmental radioactivity and its interaction with various atmospheric conditions. Both gamma and alpha radiation are significant components of the natural radiation background. They are affected by factors such as altitude, geological bedrock, soil composition and weather conditions^1–5^.

Considering weather conditions, one of the most significant and well-recognised factors influencing gamma radiation is precipitation. The increase in gamma radiation results mainly from the wet deposition of the radon progeny, particularly Pb-214 and Bi-214. Typically, gamma radiation peaks are characterised by a rapid initial increase followed by a more gradual decline. This pattern is attributed to the direct deposition of Pb-214 and Bi-214 on the ground, followed by their subsequent decay. Gamma radiation levels remain elevated above background values for several half-lives, approximately 3 to 4 h^6–8^.

The other important factor which influences gamma and alpha radiation is soil moisture, which is indirectly correlated with climate, season, weather conditions and soil composition^9–13^. When soil moisture is high for more extended periods, the gamma radiation levels diminish due to decreasing gas diffusion rates. Conversely, the reduced soil moisture content facilitated a greater level of radio-emission, as gases move upward, carrying radionuclides with them and thereby increasing ionisation near the soil surface^14^. According to Dueñas^15^, the exhalation rate of radioactive gases would be favoured by high soil permeability.

Monitoring environmental gamma and alpha radiation is essential for assessing the exposure of both the population and the environment to natural and artificial ionising radiation. Furthermore, research into variations in the natural gamma and alpha background is crucial for accurately distinguishing between meteorologically induced natural fluctuations of the outdoor absorbed dose rate in air and the increased radiation caused by artificial contamination.

The presented research aims to analyse hourly, daily and seasonal variations of gamma dose rate and alpha-emitting radionuclides across various locations to determine whether these variations exhibit similar patterns in each area. Additionally, the study seeks to evaluate the potential relationship between seasonal and daily fluctuations in gamma dose rate and alpha-emitting radionuclides and changes in weather conditions such as temperature, atmospheric pressure, wind speed and direction.

## Materials and methods

**Fig. 1:**
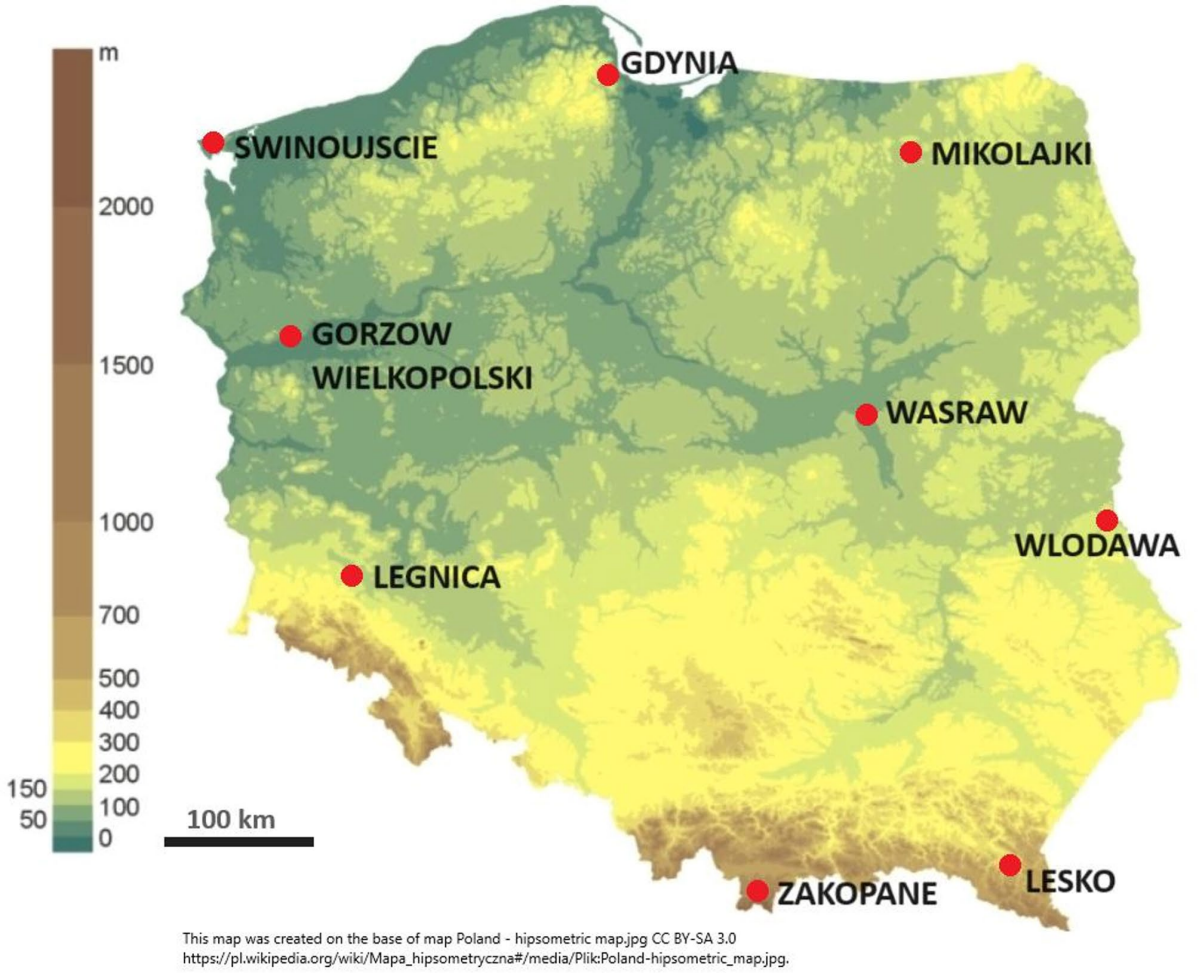
The location of the nine measurement stations.

The measurements of gamma and alpha radiation were performed at nine stations (Swinujscie, Gdynia, Mikolajki, Gorzow Wielkopolski, Warsaw, Legnica, Wlodowa, Lesko and Zakopane) in Poland belonging to the Polish Institute of Meteorology and Water Management – National Research Institute (Fig. 1).

Swinoujscie is situated on the Swina channel, which connects the northern part with the Baltic Sea and the southern part with the Szczecin Lagoon. It lies on three inhabited islands: Uznam, Wolin, and Karsibor, and 41 smaller islands that are uninhabited. Slight temperature fluctuations between day and night and mild winter temperatures characterise the maritime climate of this area. The annual precipitation is approximately 600 mm, and the humidity is variable. This area experiences the highest frequency of westerly air currents in Poland. In summer, these winds are cold and bring rainfall, while in winter, they cause thaws, which help moderate the climate. The second factor shaping the climate is the proximity of the Baltic Sea. Autumn and winter are warmer here than outside its influence, while summer and spring are colder. Considering geological structure, Swnoujscie is located in the Mid-Polish Trough zone in its northern segment – the Szczecin Basin. The entire area is covered by Quaternary sediments, Pleistocene and Holocene^16^.

Gdynia is situated on the Baltic Sea, on the Gdansk Coast and the Eastern Pomeranian Lake District, and is part of the Tricity (along with Gdansk and Sopot). The city is in a temperate climate zone, transitional between oceanic and continental climates modified by the Baltic Sea’s direct proximity. Air masses from the Gulf of Gdansk directly impact the lower layers of the atmosphere, moderating winter temperatures, lowering summer temperatures, and maintaining high humidity levels throughout the year. This region’s average annual air temperature is 7.5 °C, with precipitation averaging around 600 mm yearly. Typical for this area are strong winds, which occur in the coastal region. Considering the geological structure, Gdynia is located in the western part of the Peribaltic Depression. The entire area is covered by Quaternary sediments, Pleistocene and Holocene, with highly variable thicknesses ranging from a few to several dozen metres^17^.

Mikolajki is located in the Masurian region, within the Land of the Great Masurian Lakes, by the lakes: Talty and Lake Mikolajskie. The average annual air temperature here is approximately 6.5 °C. The average air temperature in July is about 1–2 °C lower than in central Poland and approximately 3–4 °C lower than in western Poland. The entire Masurian region is where Atlantic and continental air masses meet. The large number of open water bodies and wetlands causes the seasons to arrive later in this region compared to other parts of the country. Spring is relatively cool. Autumn is generally long and warm due to the water bodies releasing the heat accumulated during the summer. The influence of the lakes is also evident in air humidity, which fluctuates between 60% and 80% during the summer months (June–August). The annual precipitation in the Mikołajki area totals 550 mm, with the maximum occurring in June and July and the minimum in January. Winds most frequently come from the northwest and southwest, with the highest intensity in the autumn months (November–December) and early spring (March–April). Considering the geological structure, Mikolajki is located within the Precambrian East European Platform and is entirely covered by Quaternary sediments with thicknesses ranging from dozens to several hundred meters^18^.

Gorzow Wielkopolski is situated on the edge of the Gorzow Basin, located on the Gorzow Plain, along the Warta River. The city lies within an oceanic climate zone, transitioning to a continental. It is one of the warmest regions in Poland, and the annual temperature is + 8.4 °C. The climate is marked by slight yearly temperature variations, early springs, long, warm autumns, and mild winters with little snow. The region experiences relatively low annual precipitation by Polish standards. The discussed area is situated within the geostructural unit of the Gorzow Block and is covered by a continuous layer of variable thickness of Pleistocene and Holocene sediments^19^.

Warsaw is located within four mesoregions: the Warsaw Basin, the Wołomin Plain, the Middle Vistula Valley and the Warsaw Plain. The influence of both Atlantic and continental air masses creates significant weather variability throughout the year and over multi-year periods. Maritime air dominates almost two-thirds of the year, while continental masses are less frequent (22%). Like many large cities, Warsaw experiences “heat islands,” which manifest as higher average temperatures in the city centre and more frequent precipitation. Due to the increased surface roughness in the city centre, wind speeds are reduced. Considering geological structure, Warsaw is located within the Warsaw Basin. Pliocene sediments, present beneath almost the entire city, mainly consist of clays, silty and sandy muds, and sands. In many parts of the city, these sediments are exposed at the surface or are covered by a thin layer of Quaternary sediments^20^.

Legnica is located within the Silesian-Lusatian Lowland. This area is considered one of the warmest regions in the country and has a temperate climate with oceanic characteristics. The average air temperature for Legnica is 8.7 °C. The annual precipitation does not exceed 550 mm. Winds predominantly come from the west and southwest. Considering geological structure, the city lies entirely within the Fore-Sudetic Block. On top of the crystalline formations lies a layer of younger rocks from the Cenozoic era. Quaternary sediments dominate the surface, with loess covers in some parts, while Miocene sediments are exposed in certain areas^21^.

Wlodawa is situated in the Western Polesie. The area’s climatic conditions are primarily influenced by polar-maritime air masses, less frequently by polar-continental and Arctic air masses. Tropical air is the least common. The average annual temperature is 7.3 °C, which matches the national average. The area is characterised by a more extended summer season and a more prolonged winter. The average annual precipitation is around 550 mm. Considering geological structure, the city is located within the Lublin-Podlasie section of the East European Precambrian platform, within the Włodawa depression. Quaternary sediments cover the entire surface of the area, with thicknesses ranging from 9.0 to 70.9 m^22^.

Zakopane is situated in Spis–Gubałowka Highlands, the Sub-Tatra Trench, and the Tatra Mountains, along several streams that eventually flow into the Zakopianka River. It is the highest-altitude town in Poland. The average annual temperatures in this region range from 4–6ºC, corresponding to Poland’s moderately cool climate zone. Due to the downward flow of cold air masses from the mountains and the restricted air outflow from the Zakopane Basin, cold air pockets can form, with ground-level temperatures 10–20ºC lower than on the mountain slopes. This phenomenon and radiation fog negatively affect air quality, especially during the long heating season, contributing to low emissions. The average annual precipitation is 1000–1250 mm and increases with altitude, reaching 1400–2000 mm in the Tatras. A significant contribution to the water balance also comes from so-called horizontal precipitation, which is not recorded by rain gauges (condensation of fog into drizzle, rime, or ice glaze). Winds are frequent, mainly from the south or southwest in winter and from the north and south in summer. From October to May, the region experiences persistent dry and warm halny winds, reaching speeds of 25–30 m/s in the valleys and over 60 m/s on the slopes. Geologically, Zakopane lies within the Podhale Flysch region. The flysch layers, deposited during the Upper Eocene to Oligocene, comprise thick sequences of clay shales and sandstones^23^.

Lesko is located on the San River, within the Sanok-Turka Mountains. The continental climate of Eastern Europe influences the climatic conditions of the area. This is reflected in the low temperatures of the coldest month, January, and the high temperatures of the warmest month, July. The influence of warm air masses from the Hungarian plains to the south also plays a significant role. Lesko’s average annual temperature is 7.2 °C, while the total annual precipitation is approximately 750 mm. Considering geology, the city is situated within folded formations of various facies from the Krosno Layers, which belong to the Central Carpathian Synclinorium (Silesian Unit)^24^.

The Quaternary sediments are usually characterised by a low content of radionuclides – except loess cover. Loess originates from the mechanical weathering of felsic rocks such as granite and gneiss, which are naturally rich in thorium and uranium. During weathering, resistant minerals, including zircon, monazite and allanite, are concentrated in the fine-grained residue. Higher natural radionuclide contents than Quaternary sediments characterise the Oligocene clay slates or coarse-grained sandstones.

### Gamma radiation dose rate

The level of gamma radiation associated with the presence of isotopes emitting this type of radiation is determined in the atmosphere by the dose rate. The measurements of gamma radiation dose rate were performed using a TDSG probe. The TDSG probe was used to continuously measure gamma radiation dose rate in the air and distinguish dose rates originating from natural and artificial isotopes through spectrometric measurement of gamma radiation. The TDSG probe, installed approximately 1 m above the ground, simultaneously and independently measured the ambient dose equivalent rate H*(10) using a sensitive Geiger-Muller (GM) detector and the gamma radiation spectrum in the air using a NaI(Tl) scintillator detector.

The parameters of the GM counter along with electronic systems (HV, amplifiers, a system converting pulses into dose rate in nSv/h) were as follows: sensitivity of approximately 800–900 counts/min/µSv/h, measurement range of 0.01–3000 µSv/h, energy response compensated for measuring ambient dose equivalent rate H*(10) in the range of 35 keV to 1.3 MeV.

Moreover, the TDSG probe was equipped with sensors for controlling air temperature and humidity.

The scintillator detector with a NaI (Tl) crystal, was used to distinguish between dose rates from natural and artificial isotopes.

### Concentration of alpha-emitting radionuclides of natural origin

Radon decay product measurements were performed using a Berthold LB 9128 or LB 9128-MAXI monitor with a glass-fibre filter belt. The filtration tape was guided in the dust collection area on cylindrical bearings, through which air flowed. This ensured even dust accumulation with continuous and uniform filter movement without the risk of tearing the filtration tape. An alpha Si-CAM detector was placed directly above the dust collection surface, and the radioactivity of dust particles was measured. The dust collection unit and detectors were shielded with a 4π lead shield, resulting in low intrinsic radiation and low sensitivity to ambient radiation. The large dust collection surface allowed for an airflow rate of > 20 m³/hour, resulting in low detection thresholds. A multi-channel LB9000 recorder, connected to a PC-based data recorder, was used for data recording and evaluation.

### Weather conditions

The hourly and daily temperature, wind speed, and relative humidity data were downloaded from the Polish Institute of Meteorology and Water Management—National Research Institute website. The weather stations are in the same area as gamma and alpha radiation monitoring stations.

### Statistical analyses

The hourly and daily data were statistically analysed. Only the hourly and daily distributions of temperature confirmed a normal distribution. The distributions of the other parameters, including gamma and alpha radiation variabilities, did not conform to a normal distribution. Therefore, non-parametric statistical methods, such as Spearman’s rank correlation, were employed to assess the interdependence between variables. The results of statistical analyses are available in Supplementary Materials (Fig. S1–S3).

## Results and interpretations

**Fig. 2:**
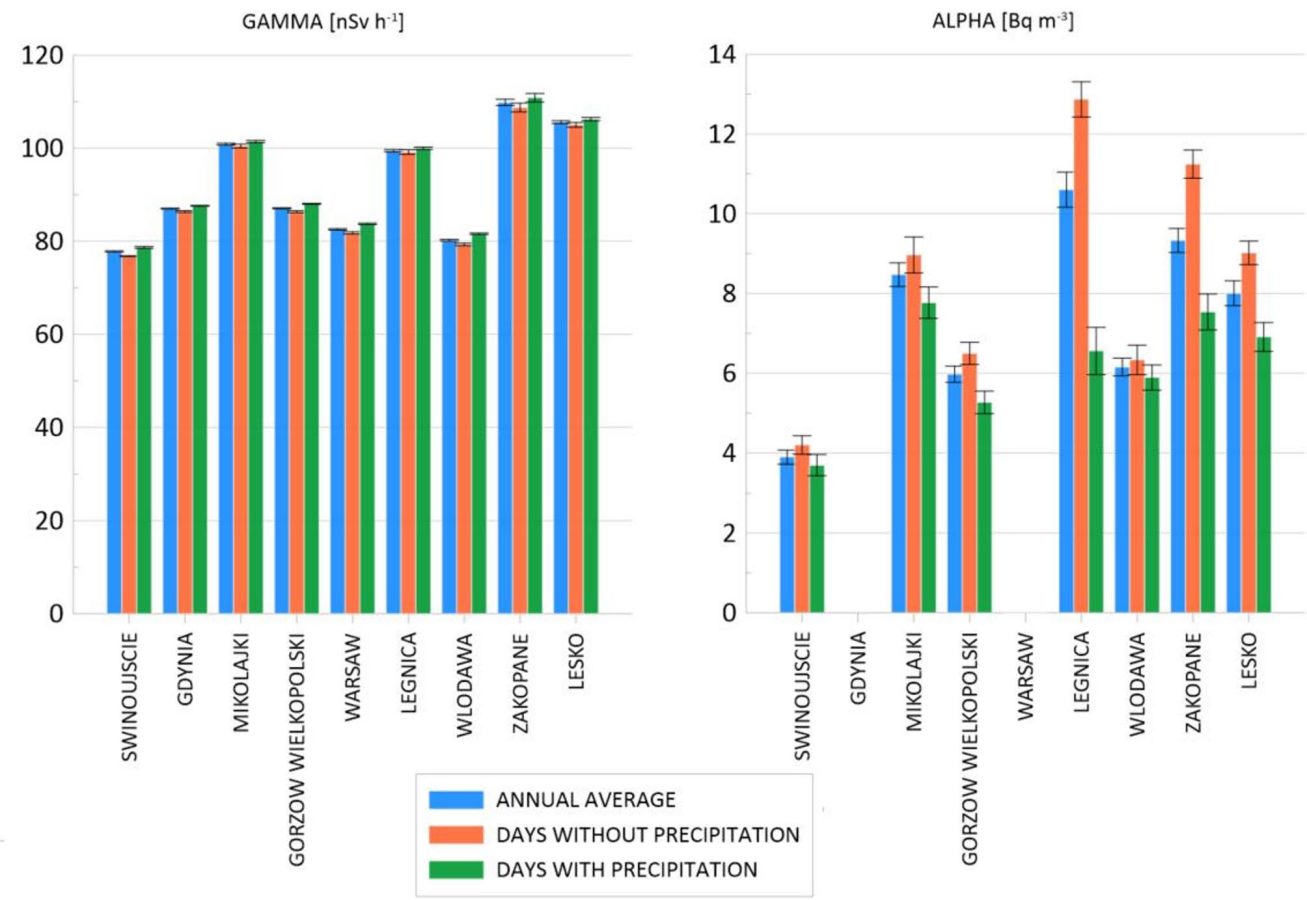
The spatial variations of annual average gamma dose rate and alpha-emitting radionuclides in nine locations.

The highest average values of gamma dose rate were recorded in Zakopane and Lesko, exceeding 100 nSy h-1, while the lowest was observed in Swinoujscie, below 80 nSv h-1. Considering the concentration of alpha-emitting radionuclides, the highest values were recorded in Legnica and the lowest in Swinujscie at levels below 4 Bq m^− 3^ (Fig. 2). The lower alpha and gamma radiation in Swinoujscie can be attributed to proximity to the sea and the presence of Quaternary sediments on the surface, which are characterised by low concentrations of naturally occurring radionuclides (U, Th, K-40). Furthermore, air masses of marine origin tend to have lower concentrations of radon and gamma-emitting radon progeny Pb-214 and Bi-214 compared to air masses of continental origin^25–27^.

The highest gamma radiation values in Zakopane and Lesko can be connected with the location of the cities at high altitudes (the Tatra Mountains and Sanok-Turka Mountains, respectively) and the geological settings. In Zakopane, at the place where the monitoring station is located, Oligocene clay slates and sandstones of the Zakopane Layers (Carpathian Flysch Belt) are exposed on the surface, whereas in Lesko Oligocene coarse-grained sandstones of the Krosno Layers (Carpathian Flysch Belt). The highest values of alpha radiation were recorded in Legnica accompanied by one of the highest values of gamma radiation. In Legnica, the monitoring station is located on Pleistocene loess and loess-like clays known for increased radioactivity compared to other Quaternary sediments.

Considering the differences in average values of gamma and alpha radiation calculated for days with and without precipitation, it can be concluded that precipitation affects the concentration of alpha-emitting radionuclides more significantly than it affects the gamma dose rate. This observation is accurate across all locations studied. The concentration of naturally occurring isotopes emitting alpha radiation is noticeably higher on days without precipitation. As radon and thoron decay in the atmosphere, their decay products accumulate in clusters and may attach to aerosol particles. After rainfall, these tiny particles suspended in the air are washed out and settled on the ground^28,29^. Moreover, rain causes an increase in soil moisture, which can reduce the exhalation of radon from the ground^30,31^. In the case of gamma radiation, the differences between rainy and non-rainy days are not significant. However, gamma radiation levels recorded on rainy days were slightly higher at each location. This may be attributed to radon decay products being washed out by the rain and settled on the ground surface, causing an increase in gamma radiation^26^. On the other hand, the increase in soil moisture causes a decrease in gamma radiation^32,33^. The co-existence of these two factors, whose effects counterbalance each other, may contribute to the milder difference observed between results calculated for rainy and non-rainy days compared to the data of alpha-emitting radionuclides.

In summary, accurate assessment of gamma and alpha radiation in the air requires careful consideration of geological and geographical factors, as these directly influence the sources, distribution, and behaviour of natural radionuclides—for example, altitude, proximity to the sea or the type of underlying rocks.

### Hourly variations in gamma radiation

The influence of meteorological conditions on hourly variations in gamma radiation was analysed for periods without precipitation because the influence of precipitation on gamma radiation is well described in the literature.

**Fig. 3:**
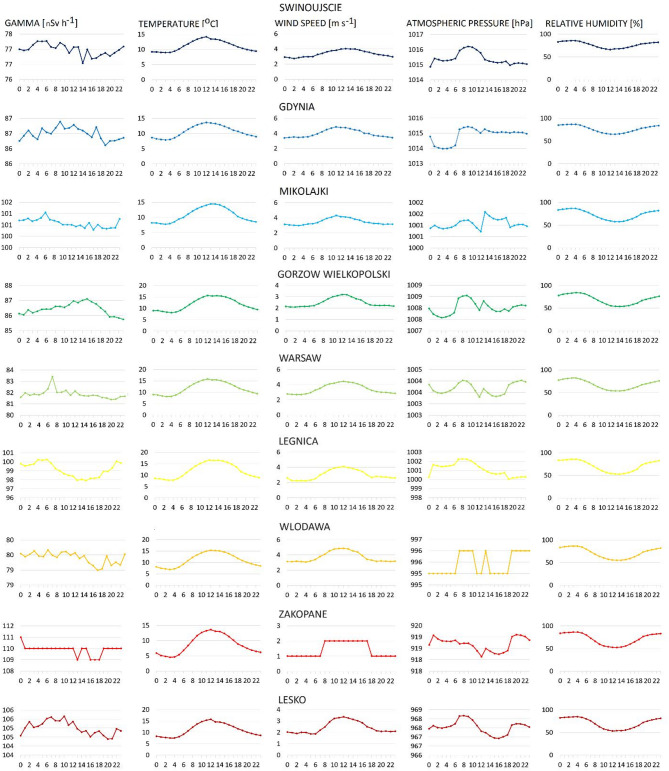
Based on yearly data, the general trend of hourly variations of gamma radiation and meteorological parameters on days without precipitations.

One typical pattern of hourly variations in gamma radiation across all locations was not observed (Fig. 3). Diurnal variations in gamma radiation are typically associated with diurnal variations in atmospheric radon concentration and nighttime temperature inversion. Therefore, gamma radiation is expected to decrease after sunrise and gradually increase after sunset^34^. In our study, a similar pattern was observed only at stations located in Legnica and Lesko.

The hourly variations in temperature, relative humidity and wind speed were similar across all studied locations. In contrast, the hourly changes in atmospheric pressure across the various locations were more complex, generally showing lower values from midnight until sunrise and higher before midday.

The statistical analysis performed on the hourly data indicates a positive correlation between gamma radiation and temperature, observed in all places except Swinujscie and Gdynia, with a Spearman correlation coefficient ranging from 0.33 to 0.55. The analysis of the relationship between atmospheric pressure and gamma radiation revealed a negative correlation in most locations, with Spearman correlation coefficient ranging from − 0.29 to -0.52, and a weak or very weak but statistically significant negative correlation in three places (Mikolajki, Lesko, Zakopane) with a correlation coefficient between − 0.12 to -0.22. The correlations between other analysed parameters, such as relative humidity or wind speed, are not obvious or statistically significant (Table 1).

**Table 1:** The values of correlation coefficients for gamma radiation and meteorological parameters at each location.

	Variable	Spearman_corr	Spearman_p
Swinoujscie	Temperature	0.09	5.98E-01
	**Wind speed**	**-0.67**	**1.26E-05**
	**Pressure**	**-0.35**	**4.22E-02**
	Relative humidity	-0.01	9.71E-01
	Gamma	0.28	1.09E-01
Mikolajki	**Temperature**	**0.40**	**4.25E-03**
	**Wind speed**	**-0.42**	**2.40E-03**
	**Pressure**	**-0.29**	**4.23E-02**
	Relative humidity	0.14	3.52E-01
	**Gamma**	**0.53**	**8.89E-05**
Gorzow Wielkopolski	Temperature	0.05	7.64E-01
	**Wind speed**	**-0.45**	**4.52E-03**
	**Pressure**	**-0.31**	**5.61E-02**
	Relative humidity	0.04	8.01E-01
	Gamma	0.27	1.02E-01
Legnica	Temperature	-0.16	2.34E-01
	**Wind speed**	**-0.65**	**6.05E-08**
	Pressure	0.07	5.84E-01
	Relative humidity	0.24	6.72E-02
	Gamma	0.19	1.59E-01
Wlodawa	**Temperature**	**0.47**	**3.39E-03**
	**Wind speed**	**-0.31**	**6.55E-02**
	**Pressure**	**-0.55**	**4.40E-04**
	**Relative humidity**	**0.47**	**3.40E-03**
	**Gamma**	**0.78**	**1.62E-08**
Zakopane	**Temperature**	**0.61**	**7.05E-04**
	**Wind speed**	**-0.65**	**1.63E-04**
	Pressure	-0.05	8.12E-01
	Relative humidity	0.20	3.01E-01
	**Gamma**	**0.67**	**9.26E-05**
Lesko	**Temperature**	**0.45**	**1.28E-02**
	Wind speed	-0.16	3.84E-01
	Pressure	-0.10	6.03E-01
	Relative humidity	0.10	6.13E-01
	**Gamma**	**0.62**	**2.47E-04**

The statistically significant values (*p* < 0.05) are marked in bold.

The temperature has a positive correlation with radioactivity: generally, high temperatures cause soil moisture to decrease and vacate the pore; thus, the dose absorbed in the air is higher^35,36^.Our results confirm this statement. The lack of correlation in Swinujscie and Gdansk may be attributed to their locations on the Polish seaside, where the sea breeze occurs (Fig. 6). The radioactivity of air from the sea is lower than that from the land, and this phenomenon could disrupt the trend observed at other stations.

The negative correlation between atmospheric pressure and outdoor gamma radiation was observed byAvdic et al., 2020^37^ in the Federation of Bosnia and Herzegovina, whereas, Abbasi research team^1^ observed a positive correlation between ambient gamma dose rate and atmospheric pressure at measurement points located on the northeast coastline of Cyprus. Our results indicate that higher atmospheric pressure reduces the gamma dose rate, probably due to reduced exhalation of radioactive gases from the ground. This relationship was less evident for stations in the mountains area, where atmospheric pressure is generally lower than in other regions.

### Daily variations in the concentration of alpha-emitting radionuclides of natural origin

**Fig. 4:**
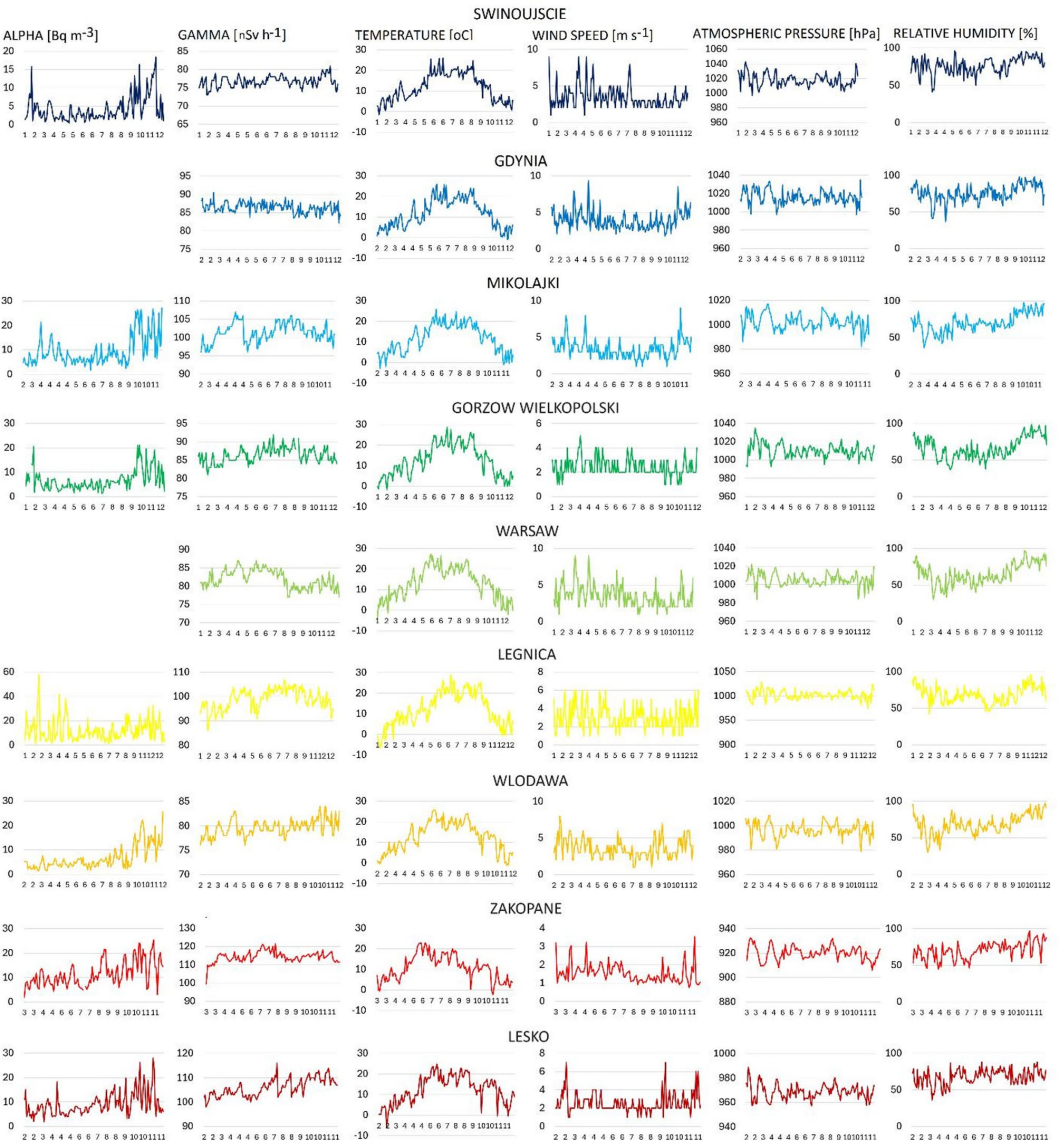
The daily variations of alpha-emitting radionuclides and meteorological parameters from January to December for days without precipitations.

The daily variations in alpha-emitting radionuclides and meteorological parameters from January to December for days without precipitation are presented in Fig. 4. Greater daily variations in alpha-emitting radionuclides were registered from September to March at almost all locations.

The relationship between daily variations in alpha-emitting naturally occurring radionuclides and weather conditions was analysed, and Spearman correlation coefficients were calculated (Table 2). A negative correlation between alpha-emitting radionuclides and atmospheric pressure was observed at almost all stations, with correlation coefficients ranging from − 0.29 to -0.61. In Lesko, Zakopane and Legnica, the negative trend was noticed, but the calculated correlation coefficients were low and not statistically significant. Lesko and Zakopane are located in the Polish mountains. In the mountains, the layer of the atmosphere pressing on the Earth’s surface is thinner, resulting in lower atmospheric pressure than in areas situated on lowlands or the coast. The registered atmospheric pressure in Zakopane and Lesko was consistently below 1000 hPa.

**Table 2:** The values of correlation coefficients for the concentration of alpha-emitting radionuclides and meteorological parameters at each location.

	Variable	Spearman_corr	Spearman_p
Swinujscie	Temperature	-0.01	4.61E-01
	Wind speed	-0.03	2.20E-02
	**Pressure**	**-0.56**	**0.00E + 00**
	Relative humidity	0.10	1.12E-17
Gdynia	Temperature	0.00	9.21E-01
	Wind speed	0.14	1.16E-32
	**Pressure**	**-0.52**	**0.00E + 00**
	Relative humidity	0.01	3.13E-01
Mikolajki	**Temperature**	**0.48**	**0.00E + 00**
	Wind speed	-0.06	1.78E-06
	**Pressure**	**-0.18**	**2.55E-50**
	Relative humidity	-0.16	1.40E-40
Gorzow Wielkopolski	**Temperature**	**0.41**	**1.07E-273**
	Wind speed	0.04	1.89E-03
	**Pressure**	**-0.46**	**0.00E + 00**
	Relative humidity	-0.16	5.06E-42
Warsaw	**Temperature**	**0.22**	**1.12E-18**
	Wind speed	-0.04	1.47E-01
	**Pressure**	**-0.21**	**4.77E-16**
	Relative humidity	0.20	2.28E-15
Legnica	**Temperature**	**0.45**	**0.00E + 00**
	Wind speed	-0.19	1.06E-60
	**Pressure**	**-0.29**	**4.92E-141**
	Relative humidity	0.00	8.89E-01
Wlodawa	**Temperature**	**0.33**	**2.06E-188**
	Wind speed	0.05	3.56E-05
	**Pressure**	**-0.37**	**7.56E-235**
	Relative humidity	0.01	3.74E-01
Zakopane	**Temperature**	**0.56**	**0.00E + 00**
	Wind speed	0.00	8.42E-01
	**Pressure**	**-0.12**	**4.90E-20**
	Relative humidity	-0.06	1.04E-05
Lesko	**Temperature**	**0.33**	**3.38E-174**
	Wind speed	0.05	1.24E-04
	**Pressure**	**-0.13**	**4.34E-26**
	Relative humidity	-0.07	7.69E-08

The statistically significant values (*p* < 0.05) are marked in bold.

Moreover, negative correlation coefficients were noticed between alpha-emitting radionuclides in the atmosphere and wind speed at all locations, with Spearman correlation coefficient ranging from − 0.31 to -0.67. Wind causes air movement, which helps disperse airborne particles, including alpha-emitting radionuclides. It also spreads these particles over a larger area, reducing their concentration at specific locations. Additionally, wind mixes the radionuclides with cleaner air, leading to a dilution effect. Furthermore, wind may push some particles towards the ground or nearby surface (such as vegetation or buildings), removing them from the air and decreasing radionuclide concentrations in the atmosphere. Considering the abovementioned factors, the observed negative relationship between alpha-emitting radionuclides and wind speed appears justified.

In addition, a positive correlation was observed between the concentration of alpha-emitting radionuclides and temperature at several stations, including Mikolajki, Wlodawa, Zakopane, and Lesko, with correlation coefficients ranging from 0.40 to 0.61. A slight positive trend was noted in Gorzow Wielkoposki, Swinujscie, where the Spearman correlation coefficients were positive but had low values and were not statistically significant. Temperature influences radon exhalation. An increase in temperature promotes the flow of radon from the ground to the atmosphere. Moreover, temperature affects soil moisture. During dry, hot conditions, the ground loses moisture, causing the soil to become loose and dusty. As a result, small soil particles can become airborne more efficiently, to which short-lived radon progeny may attach.

Analysing the relationship between the concentration of alpha-emitting radionuclides gamma radiation, a positive correlation was noticed at some stations, including Mikolajki, Włodawa, Zakopane and Lesko, with the correlation coefficients ranging from 0.53 to 0.78. A slightly positive trend was noted in Swinoujscie, Gorzow Wielkoposki, with positive Spearman correlation coefficients, but not statistically significant.

In conclusion, negative correlations were registered between daily variations in alpha-emitting radionuclides and atmospheric pressure and wind speed, and positive with temperature. The relationships between radon, thoron and their alpha-emitting decay products in the atmosphere and weather conditions are not always straightforward. The negative correlation between atmospheric pressure and radon concentration has been observed in Hungary, specifically in mofettes from Harghita and Covasna Counties^38^ as well as in the Gobi desert in northeast China^30^. Conversely, a positive correlation between atmospheric pressure and radon exhalation was recorded in volcanic areas of Italy^39^. Additionally, a positive correlation between radon concentration and temperature was observed in the Garhwal region (northeast Himalaya)^40^. In contrast, the research conducted in the southwest of the Iberian Peninsula revealed that concentrations of radon, thoron and their progenies in the atmosphere negatively correlate with temperature^41^.

### Seasonal variations in gamma radiation and concentration of alpha-emitting radionuclides

**Fig. 5:**
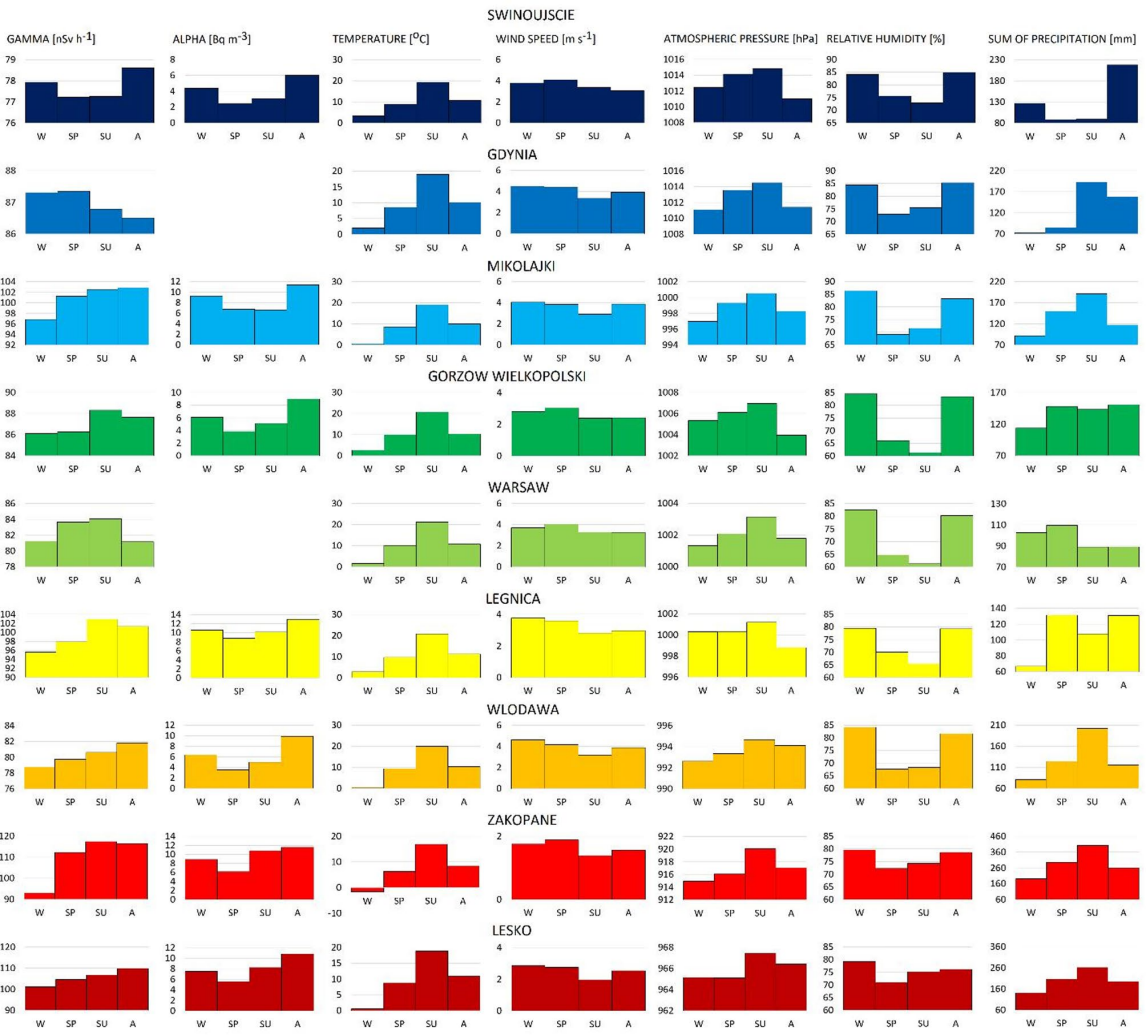
The seasonal variations in gamma radiation and alpha-emitting naturally occurring radionuclides and meteorological parameters.

The highest average values of radon progeny emitting alpha radiation were noticed during autumn and the lowest in spring in almost all studied cities (in Mikołajki, the highest values were in summer) (Fig. 5). A comparable trend of seasonal variations in radon progeny was recorded in Bucharest (Romania) with the highest values in autumn and the lowest in the spring-summer months^42^. The seasonal variations in radon and radon progenies concentrations in the atmosphere do not always follow similar patterns because local conditions impact radon exhalation from the ground and its accumulation or dilution in the air. At the uranium tailing spoil tip located in SW Sudetes, the highest radon concentrations were recorded during autumn, while the lowest in winter^43^. In India, the highest values of radon concentrations were measured in December and the lowest in June^44^ Whereas, in Canada in Montreal, the highest concentrations of radon progeny were recorded in summer and lowest in winter^45^.

**Fig. 6:**
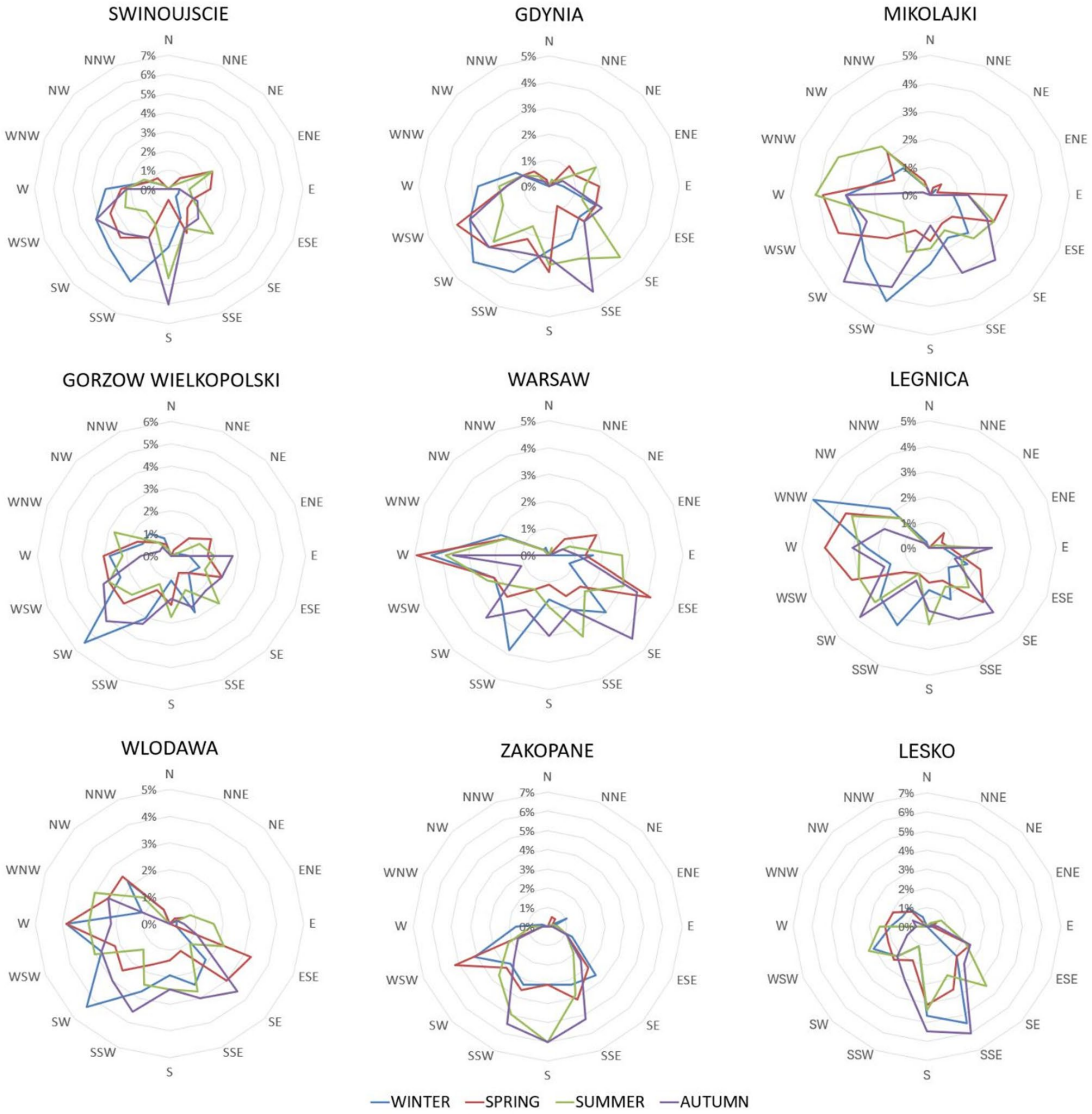
Frequency of wind directions during four seasons at nine stations.

Seasonal variations in radon and radon progeny concentrations are typically influenced by precipitation, soil moisture, changes in prevailing winds, and thermal inversion^42,44^. The seasonal variations in meteorological conditions are presented in Figs. 5 and 6. The seasonal changes in temperature and relative humidity are similar across all locations, while the seasonal variations in wind speed, wind direction and precipitation vary more significantly among locations. The strongest winds were recorded in winter (Lesko, Wlodawa, Legnica, Mikolajki, Gdynia) or in spring (Swinujscie, Gorzow Wielkopolski, Warsaw, Zakopane), and the weakest in summer in all locations except for Swinujscie, where the weakest winds occurred in autumn. The highest total precipitation was recorded in autumn (Swinoujscie, Gorzow Wielkoposki, Legnica) or summer (Lesko, Zakopane, Wlodawa, Mikolajki, Gdynia) and the lowest precipitation was recorded in winter (Lesko, Zakopane, Wlodawa, Legnica, Gorzow Wielkopolski, Mikolajow, Gdynia), or in spring and summer (Swinoujscie), or in summer and autumn (Warsaw).

It is challenging to identify a general trend in the seasonal variations of gamma radiation across all locations (Fig. 5). The highest values were recorded in summer (Gorzow Wielkopolski, Warsaw, Legnica, Zakopane), autumn (Lesko, Wlodawa, Mikolajki, Swinujscie) or in spring (Gdynia), whilst the lowest were generally observed in winter at almost all locations, except for Swinoujscie and Gdynia. Swinujscie and Gdynia are situated on the Polish seaside. Air masses from the sea (north-east and east-northeast), which occur more frequently during spring and summer, may influence the recorded gamma radiation values (Fig. 6).

According to previous research conducted in the Sudetes and Sudeten Foreland (south-west Poland), it was difficult to identify a prevailing trend in the seasonal ambient gamma dose rate; however, slightly higher values were recorded in the warmer seasons and lower values in the colder seasons^5^. In Bucharest (Romania), seasonal variations in the outdoor absorbed dose rate showed minimum values in winter and maximum values at the end of summer or the beginning of autumn^35^. In Panchkula, Haryana (India), the outdoor gamma dose rate was higher during summer and lower during winter^46^. No statistical differences between summer and winter were observed in the Balod District of Chhattisgarh (India)^47^. Conversely, examining seasonal fluctuations in ambient gamma dose rates and the associated outdoor gamma radiation risk in the northeast region of Cyprus indicated that the highest values were recorded in summer and the lowest in winter^1^.

Generally, the highest values of alpha-emitting radionuclides were observed during autumn and the lowest during spring. In contrast, for gamma radiation, the highest values were recorded in summer or autumn and the lowest in winter. The complex multi-level interrelations between weather conditions and gamma radiation and concentrations of alpha-emitting radionuclides require further in-depth analyses.

## Conclusions

The findings of research into spatial, seasonal, daily, and hourly variations in gamma radiation and alpha-emitting radionuclides indicate that geological structure, elevation above sea level and proximity to the sea, together with temperature, atmospheric pressure, and wind speed, influence the level of background radiation.

The highest annual values of gamma dose rate were recorded in the mountains, at stations where Oligence clay slates and sandstones are exposed at the surface. The lowest values of gamma radiation and alpha-emitting radionuclides were observed at the seaside stations located on Quaternary sediments. Taking into account differences in results between rainy and non-rainy periods, it may be concluded that precipitation exerts a more pronounced effect on the concentration of alpha-emitting radionuclides than on the gamma dose rate.

One typical pattern of hourly variations in gamma radiation among all locations was not observed. Statistical analyses revealed a positive correlation between gamma radiation and temperature at most sites, except Swniujscie and Gdynia, where the influence of sea breeze likely affected the recorded levels of gamma dose rate. We did not notice the statistically significant impact of other meteorological parameters, such as wind speed and atmospheric pressure, on hourly variations in gamma radiation.

Analysis of day-to-day variations in concentrations of alpha-emitting radionuclides indicated that more distinctive daily differences occurred between September and March at nearly all locations. A positive relationship or trend was observed between the concentration of naturally occurring isotopes emitting alpha radiation and temperature. The relationship between radon and thoron decay products and atmospheric pressure or wind speed was generally negative at most locations.

It is challenging to identify a general trend in the seasonal variations of gamma radiation across all locations. In the case of seasonal variations in alpha-emitting radionuclides concentrations, the highest average values were observed during autumn and the lowest in spring.

## Electronic supplementary material

Below is the link to the electronic supplementary material.

Supplementary Material 1

## Data Availability

The data will be available on request. Please contact Dagmara Tchorz-Trzeciakiewicz at: dagmara.tchorz-trzeciakiewicz@uwr.edu.pl. The statistical analyses are available in the supplementary material.

## References

[1] Abbasi, A., Zakaly, H. M. H., Bashiry, V. & Alrowaily, A. W. The seasonal variation of ambient gamma radiation dose rate and health risk assessment in North coast, mediterranean sea. *J. Radioanal Nucl. Chem.* **332**, 4355–4361 (2023).

[2] Amestoy, J. et al. Effects of environmental factors on the monitoring of environmental radioactivity by airborne gamma-ray spectrometry. *J. Environ. Radioact*. **237**, 106695 (2021). 34332827 10.1016/j.jenvrad.2021.106695

[3] Cinelli, G., Tondeur, F., Dehandschutter, B., Menneson, F. & Rincones, J. Harmonization and mapping of terrestrial gamma dose rate data in Belgium. *J. Environ. Radioact*. **248**, 106885 (2022). 35436723 10.1016/j.jenvrad.2022.106885

[4] Radumilo, V., Knežević, I. & Arbutina, D. Correlation of radiation and meteorological parameters during environmental radiation monitoring in public company Nuclear Facilities of Serbia. In *RAD Conference Proceedings* 81–84 (RAD Association, 2019).

[5] Tchorz-Trzeciakiewicz, D. E., Kozłowska, B. & Walencik-Łata, A. Seasonal variations of terrestrial gamma dose, natural radionuclides and human health. *Chemosphere* **310**, 136908 (2023). 36270528 10.1016/j.chemosphere.2022.136908

[6] Barbosa, S. M., Miranda, P. & Azevedo, E. B. Short-term variability of gamma radiation at the ARM Eastern North Atlantic facility (Azores). *J. Environ. Radioact*. **172**, 218–231 (2017). 28395155 10.1016/j.jenvrad.2017.03.027

[7] Bossew, P. et al. Cort, M. Estimating the terrestrial gamma dose rate by decomposition of the ambient dose equivalent rate. *J. Environ. Radioact*. **166**, 296–308 (2017). de. 26926960 10.1016/j.jenvrad.2016.02.013

[8] Melintescu, A. et al. Radon-222 related influence on ambient gamma dose. *J. Environ. Radioact*. **189**, 67–78 (2018). 29625370 10.1016/j.jenvrad.2018.03.012

[9] Asare, E. O., Otoo, F., Adukpo, O. K. & Opoku-Ntim, I. Assessment of soil moisture on radon levels, radon exhalation, natural radioactivity, and radiological risks in offices and laboratories in GAEC. *J. Radiat. Res. Appl. Sci.* **17**, 101014 (2024).

[10] Barbosa, S. M., Huisman, J. A. & Azevedo, E. B. Meteorological and soil surface effects in gamma radiation time series - Implications for assessment of earthquake precursors. *J. Environ. Radioact*. **195**, 72–78 (2018). 30292909 10.1016/j.jenvrad.2018.09.022

[11] Benà, E. et al. A new perspective in radon risk assessment: mapping the geological hazard as a first step to define the collective radon risk exposure. *Sci. Total Environ.* **912**, 169569 (2024). 38157905 10.1016/j.scitotenv.2023.169569

[12] Gélinas, M. & Jutras, S. Gamma radiation for the Estimation of mineral soil water content in a boreal forest. *Can. J. Soil. Sci.* **104**, 191–203 (2023).

[13] Jones, W. K. & Carroll, T. R. Error analysis of airborne gamma radiation soil moisture measurements. *Agric. Meteorol.* **28**, 19–30 (1983).

[14] Guagliardi, I. et al. Effects of source rocks, soil features and climate on natural gamma radioactivity in the Crati Valley (Calabria, Southern Italy). *Chemosphere* **150**, 97–108 (2016). 26891362 10.1016/j.chemosphere.2016.02.011

[15] Dueñas, C., Fernández, M. C., Carretero, J., Liger, E. & Pérez, M. Release of 222Rn from some soils. *Ann. Geophys.* **15**, 124–133 (1997).

[16] Heliasz, Z. & Ostaficzuk, S. Explanations to the Geoenvironmental Map of Poland 1: 50 00. Sheet: Swinoujscie. (in Polish) (2009).

[17] Gawlikowska, E., Seifert, K., Pasieczna, A., Kwecko, P. & Król, J. Explanations to the Geoenvironmental Map of Poland 1: 50 00. Sheet: Gdynia. (in Polish) (2009).

[18] Dusza-Dobek, A., Bojakowska, I., Kwecko, P., Szyborska-Kaszycka, J. & Tomassi-Morawiec, H. Król J. Explanations to the Geoenvironmental Map of Poland 1: 50 00. Sheet: Mikolajki. (in Polish) (2011).

[19] Krzak, I. et al. Explanations to the Geoenvironmental Map of Poland 1: 50 00. Sheet: Santok. (in Polish) (2006).

[20] Krogulec, E. et al. *Explanations To the Geoenvironmental Map of Poland 1: 50 00* (Warszawa Wschod, 2010). (in Polish).

[21] Kochanowska, J. et al. Explanations to the Geoenvironmental Map of Poland 1: 50 00. Sheet: Legnica. (in Polish) (2004).

[22] Grędysa, A., Bojakowska, I., Kwecko, P., Miecznik, J. & Marszałek, S. Explanations to the Geoenvironmental Map of Poland 1: 50 00. Sheet: Wlodawa. (in Polish) (2011).

[23] Paulo, A. et al. *Explanations To the Geoenvironmental Map of Poland 1: 50 00* (Tatry Zachodnie, 2004). (in Polish).

[24] Wojtyna, H., Gabryś-Godlewska, A., Kwecko, P. & Tomasi-Morawiec, H. Explanations to the Geoenvironmental Map of Poland 1: 50 00. Sheet: Lesko (in Polish) (2007).

[25] Inomata, Y., Chiba, M., Igarashi, Y., Aoyama, M. & Hirose, K. Seasonal and Spatial variations of enhanced gamma ray dose rates derived from 222Rn progeny during precipitation in Japan. *Atmos. Environ.* **41**, 8043–8057 (2007).

[26] Mercier, J. F. et al. Increased environmental gamma-ray dose rate during precipitation: a strong correlation with contributing air mass. *J. Environ. Radioact*. **100**, 527–533 (2009). 19403214 10.1016/j.jenvrad.2009.03.002

[27] Paatero, J. Wet deposition of Radon-222 progeny in Northern Finland measured with an automatic precipitation gamma analyser. *Radiat. Prot. Dosim*. **87**, 273–280 (2000).

[28] Bottardi, C. et al. Rain rate and radon daughters’ activity. *Atmos. Environ.* **238**, 117728 (2020).

[29] Yakovleva, V. S., Nagorsky, P. M., Cherepnev, M. S., Kondratyeva, A. G. & Ryabkina, K. S. Effect of precipitation on the background levels of the atmospheric β- and γ-radiation. *Appl. Radiat. Isot.* **118**, 190–195 (2016). 27649028 10.1016/j.apradiso.2016.09.017

[30] Li, P., Sun, Q. & Cong, L. Study on the influence of water saturation on radon exhalation rates of rocks. *Sci. Total Environ.* **946**, 174192 (2024). 38914332 10.1016/j.scitotenv.2024.174192

[31] Yang, J. et al. Modeling of radon exhalation from soil influenced by environmental parameters. *Sci. Total Environ.* **656**, 1304–1311 (2019). 30625659 10.1016/j.scitotenv.2018.11.464

[32] De Groot, A. V., Van Der Graaf, E. R., De Meijer, R. J. & Maučec, M. Sensitivity of in-situ γ-ray spectra to soil density and water content. *Nucl Instrum. Methods Phys. Res. A*. **600**, 519–523 (2009).

[33] Filippucci, P. et al. Soil moisture as a potential variable for tracking and quantifying irrigation: A case study with proximal gamma-ray spectroscopy data. *Adv. Water Resour.* **136**, 103502 (2020).

[34] Datar, G., Vichare, G., Selvaraj, C., Bhaskar, A. & Raghav, A. Causes of the diurnal variation observed in gamma-ray spectrum using NaI (Tl) detector. *J. Atmos. Sol Terr. Phys.* **207**, 105369 (2020).

[35] Baciu, A. C. Outdoor absorbed dose rate in air in relation to airborne natural radioactivity and meteorological conditions at Bucharest (Romania). *J. Radioanal Nucl. Chem.* **268**, 3–14 (2006).

[36] Lebedyte, M., Butkus, D. & Morkunas, G. Variations of the ambient dose equivalent rate in the ground level air. *J. Environ. Radioact*. **64**, 45–57 (2003). 12469770 10.1016/s0265-931x(02)00057-7

[37] Avdic, S. et al. A study of daily variations of the outdoor background radiation measured in continuous mode in federation of Bosnia and Herzegovina. *J. Environ. Radioact*. **217**, 106212 (2020). 32217242 10.1016/j.jenvrad.2020.106212

[38] Néda, T. & Dósa, A. Seasonal variations of radon activity concentration in mofettes from Harghita and Covasna counties, Romania. *J. Environ. Radioact*. **273**, 107389 (2024). 38278089 10.1016/j.jenvrad.2024.107389

[39] Cigolini, C. et al. Radon surveys and real-time monitoring at Stromboli volcano: influence of soil temperature, atmospheric pressure and tidal forces on 222Rn degassing. *J. Volcanol Geotherm. Res.* **184**, 381–388 (2009).

[40] Kamra, L. Seasonal emanation of radon at ghuttu, Northwest himalaya: differentiation of atmospheric temperature and pressure influences. *Appl. Radiat. Isot.* **105**, 170–175 (2015). 26319089 10.1016/j.apradiso.2015.08.031

[41] Barba-Lobo, A., Gutiérrez-Álvarez, I., Adame, J. A., San Miguel, E. G. & Bolívar, J. P. Behavior of 222Rn, 220Rn and their progenies along a daily cycle for different meteorological situations: implications on atmospheric aerosol residence times and Rn daughters’ equilibrium factors. *J. Hazard. Mater.* **464**, 132998 (2024). 37988870 10.1016/j.jhazmat.2023.132998

[42] Baciu, A. C. Radon and thoron progeny concentration variability in relation to meteorological conditions at Bucharest (Romania). *J. Environ. Radioact*. **83**, 171–189 (2005). 15960998 10.1016/j.jenvrad.2005.02.015

[43] Tchorz-Trzeciakiewicz, D. E. & Solecki, A. T. Variations of radon concentration in the atmosphere. Gamma dose rate. *Atmos. Environ.* **174**, 54–65 (2018).

[44] Victor, N. J., Siingh, D., Singh, R. P., Singh, R. & Kamra, A. K. Diurnal and seasonal variations of radon (222Rn) and their dependence on soil moisture and vertical stability of the lower atmosphere at pune, India. *J. Atmos. Sol Terr. Phys.* **195**, 105118 (2019).

[45] Chah, B. & Zikovsky, L. Seasonal variation of radon daughters in outdoor air in Montreal. *Water Air Soil. Pollut*. **51**, 133–138 (1990).

[46] Tanwer, N. et al. Measurement of seasonal variation of outdoor gamma radiation dose rate level and assessment of consequent health hazards in panchkula, haryana, India. *Radiochemistry* **64**, 424–431 (2022).

[47] Jindal, M. K., Kumar, S., Shweta, S. & Arun, A. S., Risk assessment from gamma dose rate in Balod district of chhattisgarh, India. *J Radioanal Nucl. Chem* **317** (2018).

