# Supplementary material for: Variations in gamma radiation and alpha-emitting radionuclides in correlation with weather and location conditions
Source: Sci Rep. 2025 Jul 11;15:25063. doi: 10.1038/s41598-025-10797-2 (PMC12254402; doi:10.1038/s41598-025-10797-2)
Supplement: Supplementary file 1 — Supplementary Material 1 [file 41598_2025_10797_MOESM1_ESM.docx]

| Alpha  [Bq ,-3] | Gamma  [nSv h-1] | Temperature [$˚C$] | Wind speed [ms^-1^] | Air pressure [hPa] | Relative humidity [%] |
| --- | --- | --- | --- | --- | --- |
| Swinoujscie | | | | | |
| 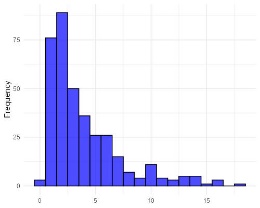 | 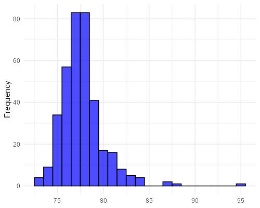 | 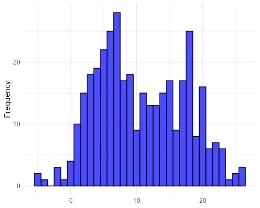 | 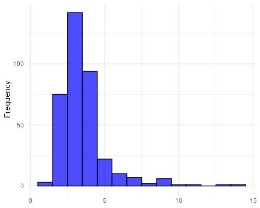 | 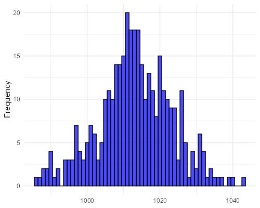 | 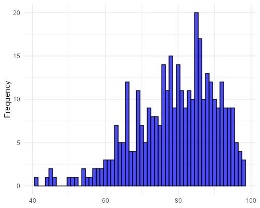 |
| Gdynia | | | | | |
|  | 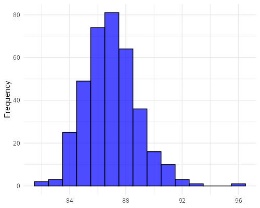 | 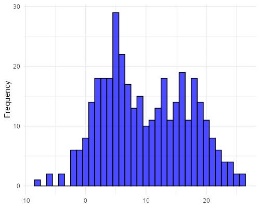 | 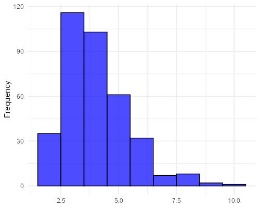 | 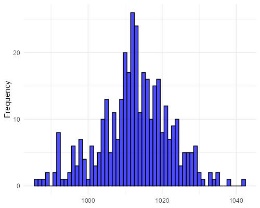 |  |
| Mikolajki | | | | | |
| 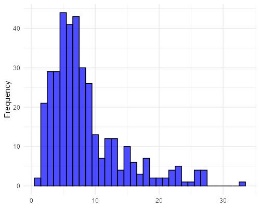 | 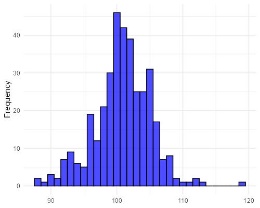 | 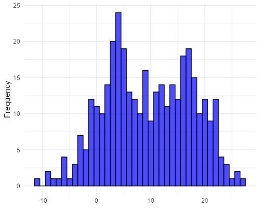 | 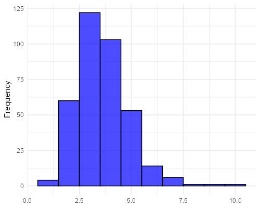 | 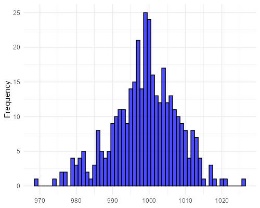 | 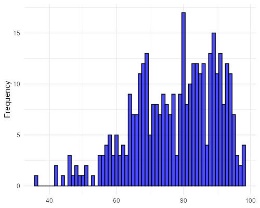 |
| Gorzow Wielkopolski | | | | | |
| 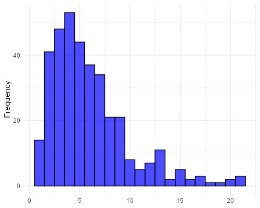 | 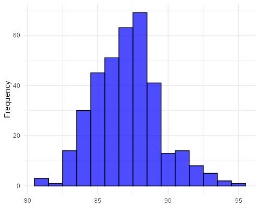 | 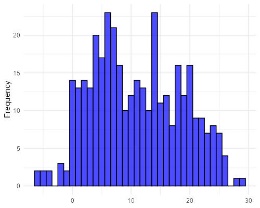 | 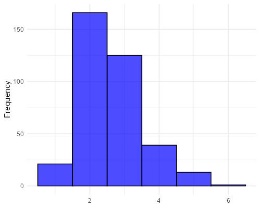 | 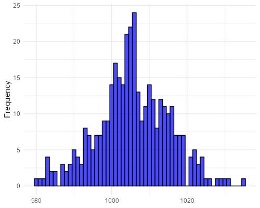 | 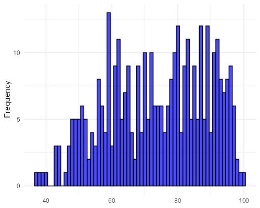 |
| Warszawa | | | | | |
| 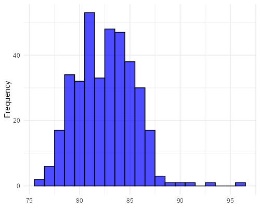 | 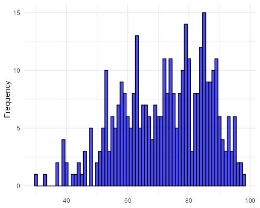 | 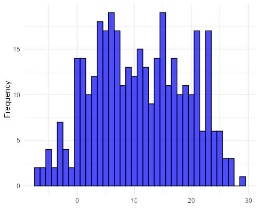 | 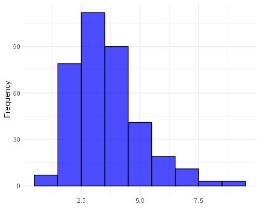 | 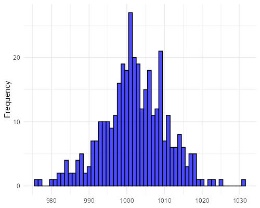 |  |
| Legnica | | | | | |
| 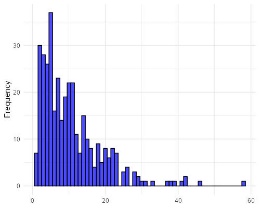 | 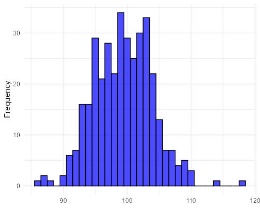 | 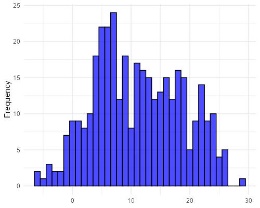 | 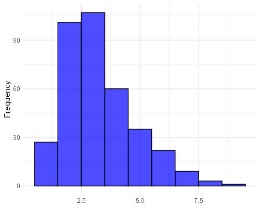 | 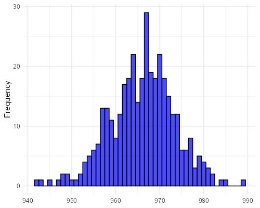 | 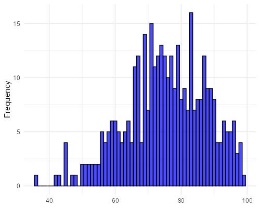 |
| Wlodawa | | | | | |
| 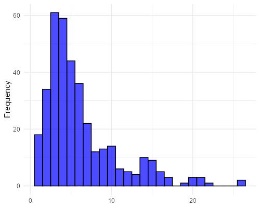 | 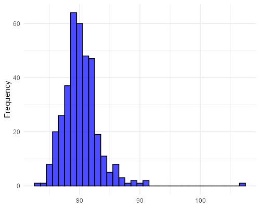 | 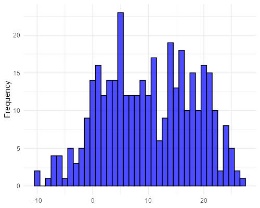 | 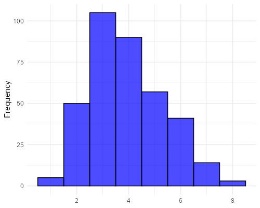 | 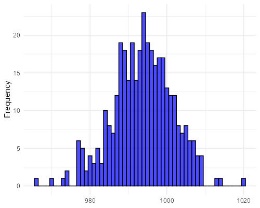 | 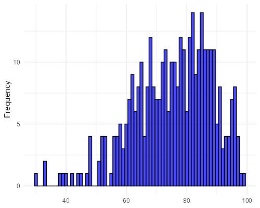 |
| Zakopane | | | | | |
| 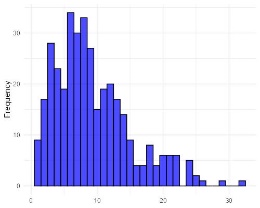 | 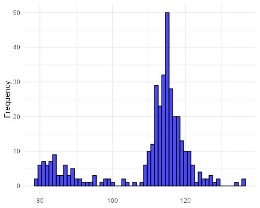 | 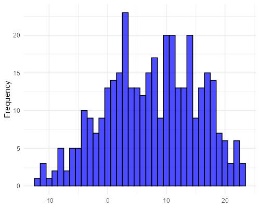 | 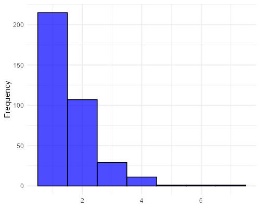 | 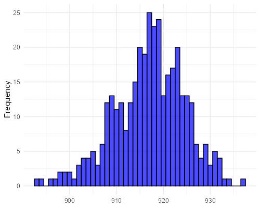 | 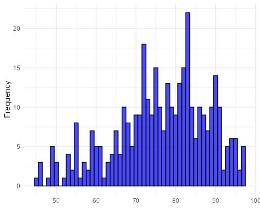 |
| Lesko | | | | | |
| 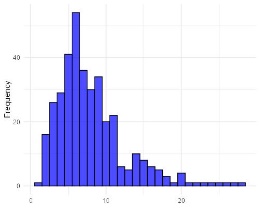 | 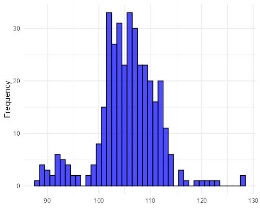 | 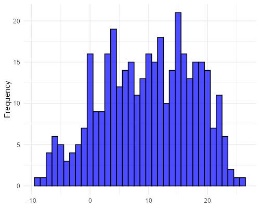 | 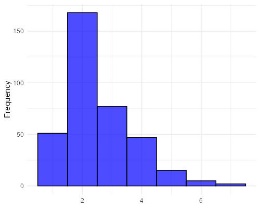 | 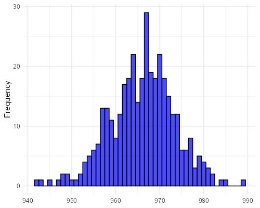 | 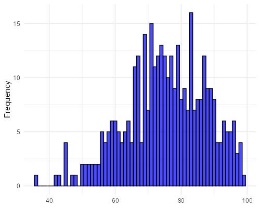 |

Figure S1. Histogram distribution of daily variations of alpha-emitting radionuclide concentrations, gamma dose rates, and meteorological parameters.

| Swinoujscie | | | |
| --- | --- | --- | --- |
| Temperature [$˚C$] | Wind speed [ms^-1^] | Air pressure [hPa] | Relative humidity [%] |
| 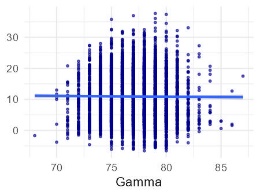 | 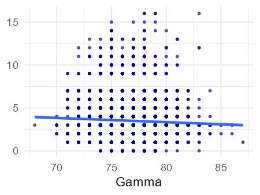 | 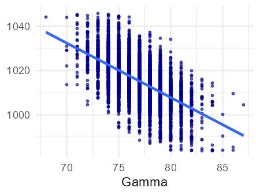 | 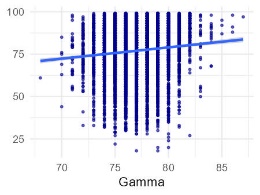 |
| Gdynia | | | |
| 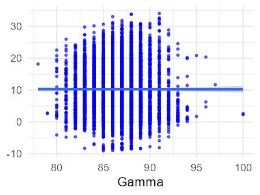 | 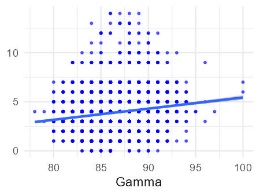 | 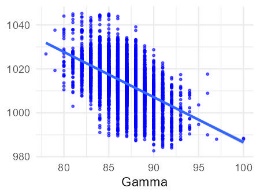 | 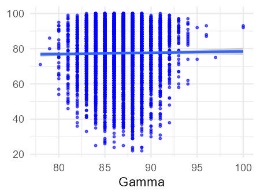 |
| Mikolajki | | | |
| 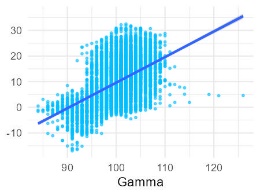 | 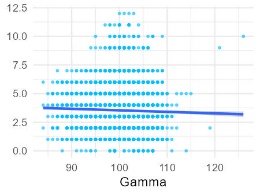 | 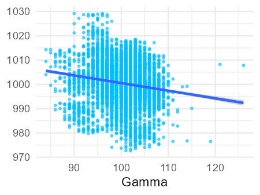 | 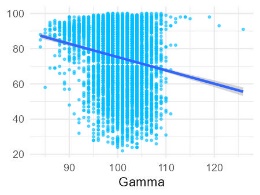 |
| Gorzow Wielkopolski | | | |
| 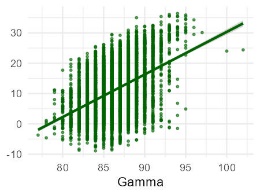 | 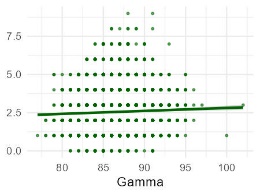 | 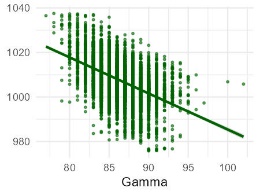 | 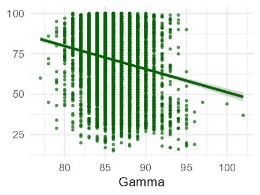 |
| Warsaw | | | |
| 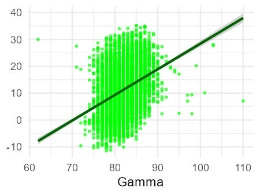 | 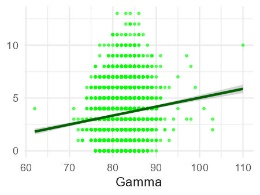 | 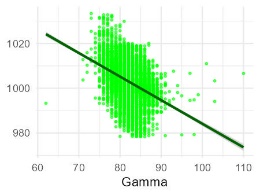 | 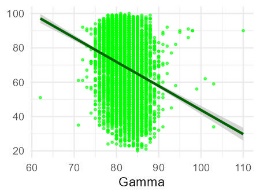 |
| Legnica | | | |
| 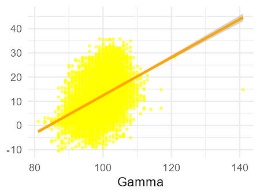 | 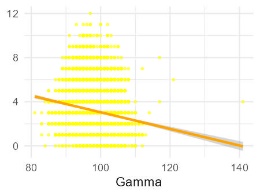 | 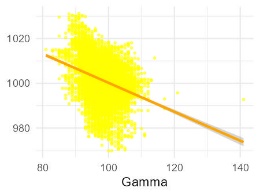 | 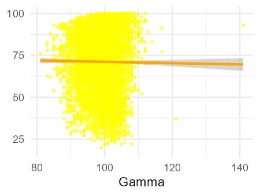 |
| Wlodawa | | | |
| 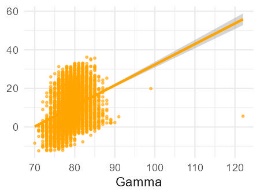 | 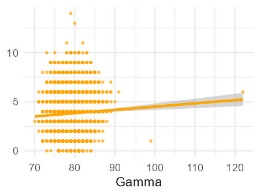 | 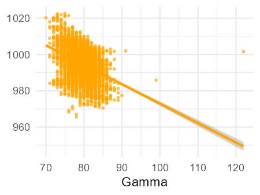 | 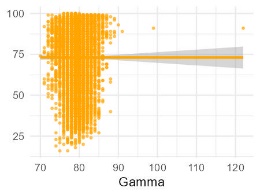 |
| Zakopane | | | |
| 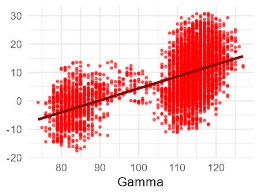 | 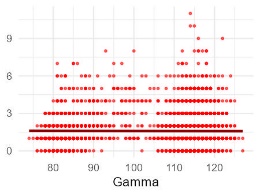 | 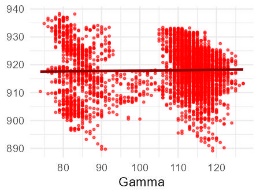 | 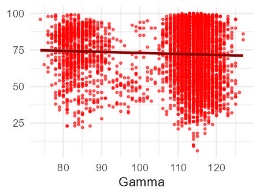 |
| Lesko | | | |
| 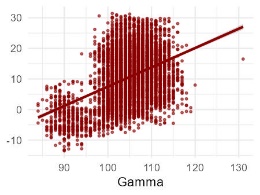 | 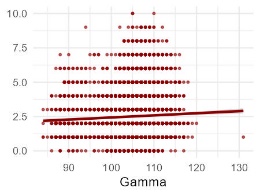 | 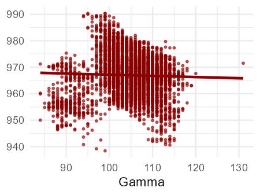 | 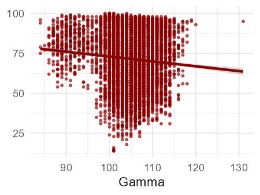 |

Figure S2. The correlation between gamma radiation and meteorological parameters at each location. The graphs with statistically significant values (p<0.05) are framed.

| Swinoujscie | | | |
| --- | --- | --- | --- |
| Temperature [$˚C$] | Wind speed [ms^-1^] | Air pressure [hPa] | Relative humidity [%] |
| 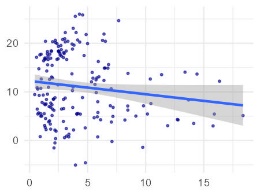 | 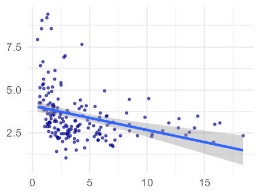 | 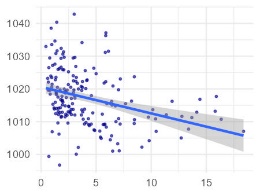 | 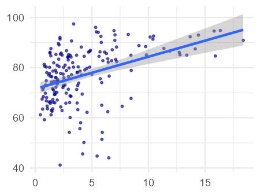 |
| Mikolajki | | | |
| 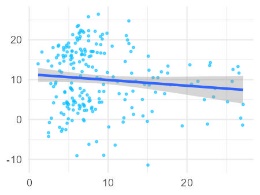 | 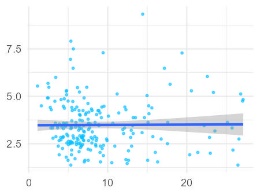 | 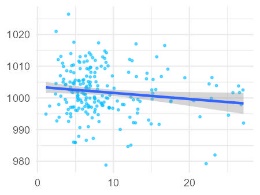 | 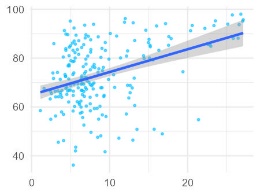 |
| Gorzow Wielkopolski | | | |
| 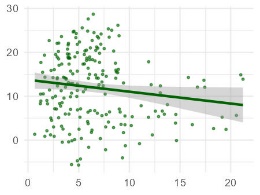 | 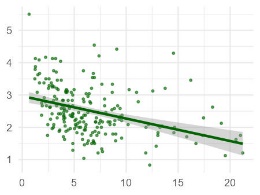 | 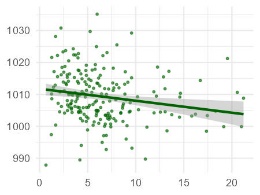 | 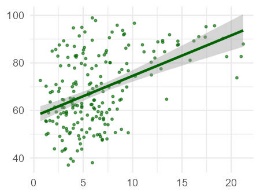 |
| Legnica | | | |
| 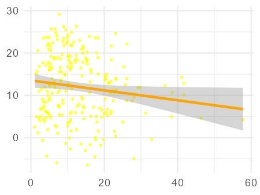 |  |  |  |
| Wlodawa | | | |
|  |  |  |  |
| Zakopane | | | |
|  |  |  |  |
| Lesko | | | |
|  |  |  |  |

Figure S3. The correlation between alpha-emitting radionuclide concentrations and meteorological parameters at each location. The graphs with statistically significant values (p<0.05) are framed.
